# Trends and Disparities in Appendicitis‐Related Mortality Across U.S. Demographics and Regions: A 22‐Year CDC WONDER Database Study

**DOI:** 10.1002/wjs.70023

**Published:** 2025-07-28

**Authors:** Rayyan Nabi, Shree Rath, Sohaib Aftab Ahmad Chaudhry, Najaf Ahmed Rajpar, Sabahat Ul Ain Munir Abbasi, Tabeer Zahid, Raheel Ahmed

**Affiliations:** ^1^ Islamic International Medical College Rawalpindi Pakistan; ^2^ All India Institute of Medical Sciences Bhubaneswar India; ^3^ ABWA Medical College Faisalabad Pakistan; ^4^ Bilawal Medical College, Liaquat University of Medical and Health Sciences Jamshoro Pakistan; ^5^ Allama Iqbal Medical College Lahore Pakistan; ^6^ Foundation University Medical College Islamabad Pakistan; ^7^ National Heart and Lung Institute Imperial College London United Kingdom London UK

**Keywords:** appendicitis, CDC wonder, emergency care, surgery

## Abstract

**Background:**

Appendicitis remains a common surgical emergency with potentially fatal complications. Long‐term trends in mortality disparities are not well characterized. We analyzed demographic and regional disparities in appendicitis‐related mortality in the United States from 1999 to 2020 using CDC WONDER data.

**Methods:**

We performed a retrospective analysis of death‐certificate data for individuals aged ≥ 25 years, identifying appendicitis‐related deaths (ICD‐10 K35–K37). Age‐adjusted mortality rates (AAMRs) per 100,000 population were standardized to the 2000 U.S. standard. Joinpoint regression estimated annual percentage changes (APCs) and average APCs (AAPCs) with 95% confidence intervals. Analyses were stratified by sex, race/ethnicity, urbanization, and state.

**Results:**

From 1999 to 2020, 15,243 appendicitis‐related deaths occurred (6643 females and 8600 males). Overall AAMR declined from 0.38 to 0.32 per 100,000 (AAPC –1.38% and 95% CI –3.10 to 0.36). A significant decrease occurred from 1999 to 2018 (APC–2.81% and *p* < 0.0001), followed by a nonsignificant rise. Males exhibited a consistent decline (AAPC –2.88% and *p* < 0.0001), whereas females experienced an increase from 2016 to 2020 (APC +5.94% and *p* < 0.0001). Black individuals had the highest AAMR (0.38) but a significant decline (AAPC –3.73% and *p* < 0.0001) compared to Whites (AAPC –2.49% and *p* < 0.0001) and Hispanics (AAPC –2.06% and *p* < 0.0001). Nonmetropolitan areas had higher AAMR (0.34) than metropolitan areas (0.31). Vermont and New Mexico recorded the highest state‐level AAMRs; New Jersey and Louisiana had the lowest.

**Conclusions:**

Appendicitis‐related mortality in the United States declined over two decades; however, rising mortality among females since 2016 and persistent racial and regional disparities underscore the need for targeted interventions to ensure equitable access to timely surgical care.

## Graphical abstract

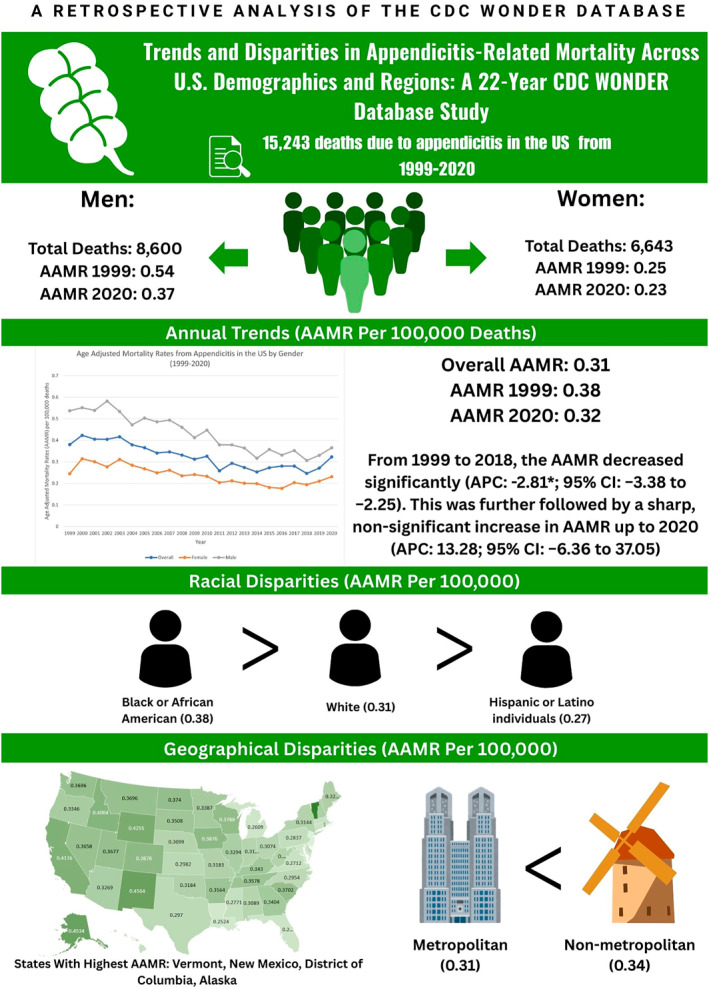

## 1 Introduction

Appendicitis is an inflammatory condition of the vermiform appendix that, if untreated, can lead to complications such as perforation, abscess formation, ileus, peritonitis, or mortality [1]. It remains one of the leading causes of abdominal emergencies requiring surgical intervention [2]. According to the 2020 WSES Jerusalem guidelines, first‐line treatment for uncomplicated appendicitis includes antibiotics; however, patients who decline surgery are informed of a 39% risk of recurrence within 5 years [3]. For both uncomplicated and complicated appendicitis, appendectomy remains the preferred treatment, with laparoscopic surgery demonstrating superior outcomes compared to open appendectomy [3].

Key estimates from the Global Burden of Disease Study 2021 indicate that the global age‐standardized mortality rate for appendicitis was 0.358 per 100,000, whereas the incidence rate reached 214 per 100,000, corresponding to 17 million new cases [4]. Between 1990 and 2021, global rates of mortality, incidence, years of life lost (YLLs), years lived with disability (YLDs), and disability‐adjusted life years (DALYs) steadily declined, with the most significant reductions seen in mortality and YLL rates [4]. Despite this global progress, substantial geographical disparities persist, highlighting inequities in access to quality healthcare [5]. Acute appendicitis is time‐sensitive, and delays in treatment increase the risk of perforation and prolonged hospital stays; therefore, appendiceal perforation rates are used as an indicator for access to surgical care [6]. A national study on perforated appendicitis admission rates (PAAR) in the United States found that younger minority adults (aged 18–34) had significantly higher rates of perforation than non‐Hispanic White patients, partly due to differences in insurance and income [7]. These disparities were not observed in populations with universal insurance coverage, underscoring the critical role of healthcare access [8, 9].

Although previous studies have explored related factors to identify limitations in care [10, 11, 12], no existing data analyses recent long‐term trends in demographic and regional disparities in appendicitis‐related mortality. Given that appendicitis‐related hospitalizations accounts for over $3 billion in annual health expenditures within the United States alone and carries a one in 15 lifetime risk [13], it becomes increasingly important to understand these patterns. Therefore, we conducted a retrospective analysis using data from the CDC Wonder (Centers for Disease Control and Prevention Wide‐Ranging Online Data for Epidemiological Research) database, examining the impact of sex, race, urbanization, state, and place of death on mortality rates. These insights can help generate hypotheses about vulnerable populations who may face limited access to timely surgical care, alert healthcare providers, guide targeted health policies and resource allocation, and promote equitable outcomes for a condition that is largely preventable through early intervention.

## 2 Methods

### 2.1 Study Design and Setting

This study is a retrospective analysis of demographic and regional disparities in mortality related to appendicitis in the United States from 1999 to 2020. Data were retrieved from the CDC Wide‐Ranging Online Data for Epidemiologic Research (CDC WONDER), which aggregates death certificate information provided by the National Center for Health Statistics (NCHS) [14]. It includes data from all 50 states and the District of Columbia about both underlying and contributing causes of death, along with demographic and geographic variables. Appendicitis‐related deaths for individuals aged 25 years or above were identified using the Multiple Cause of Death Public Use Record and International Classification of Diseases, 10th Revision, Clinical Modification (ICD‐10) codes: **K35–K37** as used by the older studies to enhance the reliability of our findings [5, 15, 16]. Our study adheres to the Strengthening the Reporting of Observational Studies in Epidemiology (STROBE) guidelines [17]. We did not require the Institutional Review Board's approval as only publicly available, anonymous patient data were used.

### 2.2 Data Extraction

Our study focuses on individuals aged 25 years and older, grouped by standard age categories up to 85 years and above. Mortality rates were calculated per 100,000 populations. In addition to the overall appendicitis‐related AAMR, data were extracted for the following variables: sex (male and female), race/ethnicity (non‐Hispanic White, non‐Hispanic Black or African American, or Hispanic or Latino), and urbanization (metropolitan and nonmetropolitan) based on the National Center for Health Statistics Urban‐Rural Classification Scheme (2013) [18] and geographic regions based on the U.S. Census Bureau definitions [19].

#### 2.2.1 Statistical Analysis

We analyzed crude mortality rates (CMRs) and age‐adjusted mortality rates (AAMRs) for appendicitis‐related deaths per 100,000 population from 1999 to 2000. To calculate the CMRs, we divided the total number of deaths by the corresponding U.S. population each year. AAMRs, on the other hand, were standardized to the 2000 U.S. standard population, to draw comparisons across subgroups over time with 95% confidence intervals (CIs) [20]. To analyze the temporal trends, we applied Joinpoint regression analysis (version 5.1.0, National Cancer Institute) to estimate annual percentage changes (APCs) with 95% CIs [21]. Any statistically significant changes (expressed by a *p*‐value < 0.05) in mortality trends over time were observed through log‐linear regression models where the slope represented the change in mortality, and its deviation from zero represented any increase or decrease in mortality, as determined by 2‐tailed t‐tests. We also performed sensitivity analyses to compare cases where appendicitis was the underlying cause versus a contributing cause of death, to get a better understanding of its role in mortality.

## 3 Results

From 1999 to 2020, there were a total of 15,243 deaths due to appendicitis in the United States among individuals aged 25 years to over 85 years. Of these, 6643 occurred in females whereas 8600 occurred in males. The results were categorized by year, sex, race, urbanization, place of death, and state.

### 3.1 Overall Appendicitis‐Related AAMR

**FIGURE 1:**
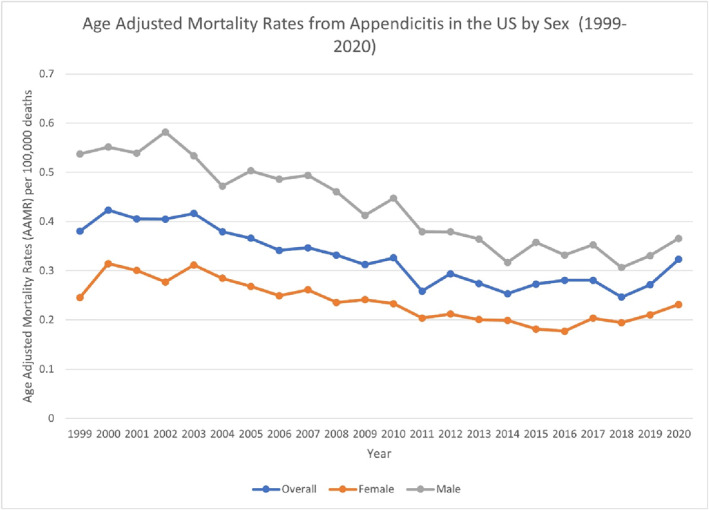
AAMR‐related to appendicitis in the United States by sex, 1999–2020.

From 1999 to 2018, an annual percentage change (APC) was −2.8119* (95% CI: −3.3754 to −2.2451 and *p* < 0.000001), indicating a significant decreasing trend for age‐adjusted mortality rate (AAMR) related to appendicitis. Between 2018 and 2020, it rose to 13.2848 (95% CI: −6.3607–37.0519). Across the entire period, the AAMR decreased from 0.38 (95% CI: 0.3507–0.4093) in 1999 to 0.3229 (95% CI: 0.2996–0.3461) in 2020, resulting in an average annual percentage change (AAPC) of −1.3829 (95% CI: −3.0980 to 0.3625) (Figure 1).

Throughout the timespan, males consistently exhibited a higher AAMR (0.3074) as compared to females (0.2282). For females, from 1999 to 2001, the APC was 9.5788 (95% CI: −4.7889–26.1146). It then decreased significantly from 2001 to 2016 at −3.5793* (95% CI: −4.2239 to −2.9304 and *p* < 0.000001) and rose significantly from 2016 to 2020 at 5.9360* (95% CI: 1.5893 to 10.4687 and *p* < 0.000001). Over the full span (1999–2020), the AAPC was −0.6320 (95% CI: −2.1015 to 0.8596). For males, from 1999 to 2020, the rates decreased linearly resulting in an AAPC of −2.8754* (95% CI: −3.3660 to −2.3823 and *p* < 0.000001), reflecting a significant decreasing trend (Figure 1) (Supporting Information S1: Table S1) (Supporting Information S1: Figure S1).

### 3.2 Appendicitis‐Related AAMR Stratified by Race/Ethnicity

**FIGURE 2:**
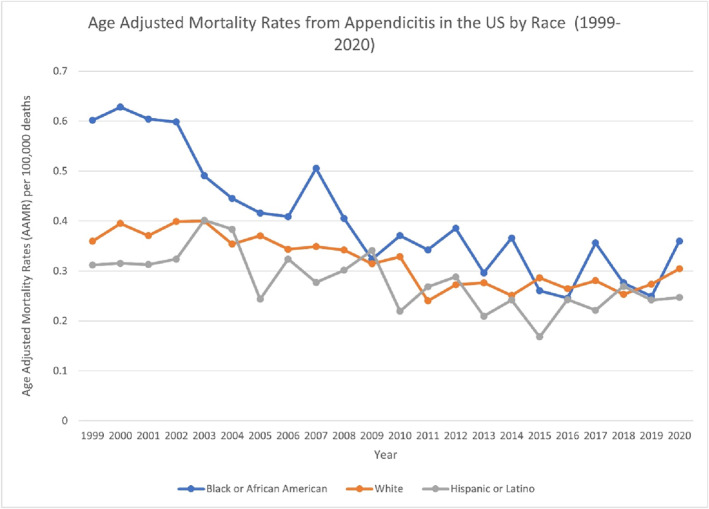
AAMR‐related to appendicitis in the United States by race, 1999–2020.

From 1999 to 2020, Black or African American individuals consistently exhibited the highest AAMR (0.3823), followed by White individuals (0.3098) and for Hispanic or Latino individuals (0.269). Black or African American population exhibited a single‐segment linear decline with an AAPC of −3.7330* (95% CI: −4.6758 to −2.7809 and *p* < 0.000001), indicating a significant decreasing trend. Among White individuals, the APC was −2.4852* (95% CI: −3.1831 to −1.7823 and *p* < 0.000001) from 1999 to 2018, a significant declining trend. From 2018 to 2020, it shifted to 9.8084 (95% CI: −13.0749–38.7158), which is not significant. Across the entire period (1999–2020), the AAPC was −1.3763 (95% CI: −3.4778 to 0.7710). For Hispanic or Latino individuals, a single‐segment APC of −2.0597* (95% CI: −3.1206 to −0.9872 and *p* < 0.000001) was observed between 1999 and 2020, representing a significant declining trend. Because it is a single segment, the AAPC also equals −2.0597* (95% CI: −3.1206 to −0.9872 and *p* < 0.000001) (Figure 2) (Supporting Information S1: Table S2) (Supporting Information S1: Figure S2). Results for American Indians/Alaska Natives and Asians/Pacific Islanders were omitted due to unreliable data.

### 3.3 Appendicitis‐Related AAMR Stratified by Urbanization Status

**FIGURE 3:**
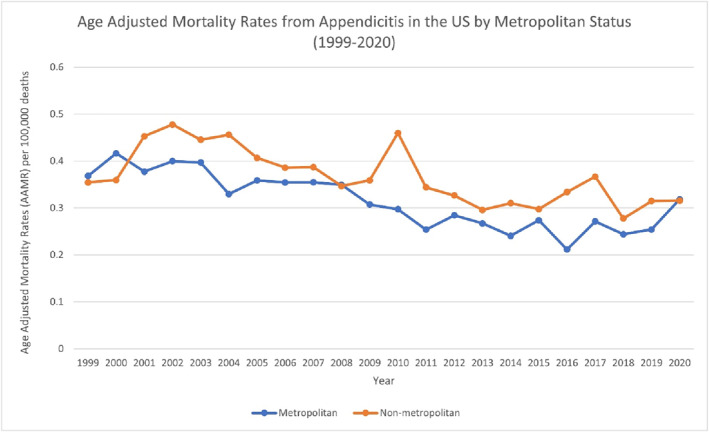
AAMR‐related to appendicitis in the United States by metropolitan status, 1999–2020.

Nonmetropolitan areas exhibited a higher AAMR (0.3435) than metropolitan areas (0.305). In nonmetropolitan areas, there was a single segment from 1999 to 2020, with an APC of −1.7681* (95% CI: −2.5773 to −0.9522 and *p* < 0.000001), a significant decreasing trend. Because only one segment is present, the AAPC also equals −1.7681* (95% CI: −2.5773 to −0.9522 and *p* < 0.000001). From 1999 to 2018, metropolitan areas recorded an APC of −2.9962* (95% CI: −3.7091 to −2.2781 and *p* < 0.000001), indicating a significant decreasing trend. Between 2018 and 2020, it shifted to 15.1695 (95% CI: −9.5499–46.6446), which is not significant. Across the entire period (1999–2020), the AAPC was −1.3974 (95% CI: −3.5677 to 0.8218) (Figure 3) (Supporting Information S1: Table S3) (Supporting Information S1: Figure S3).

### 3.4 Appendicitis‐Related AAMR Stratified by States

**FIGURE 4:**
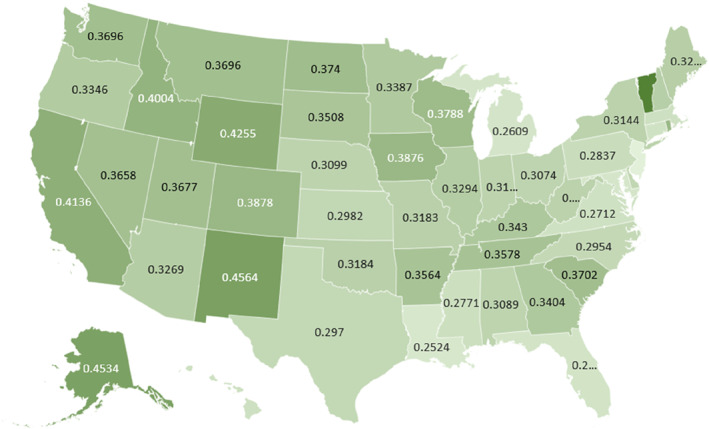
AAMR‐related to appendicitis in the United States by state, 1999–2020.

Between 1999 and 2020, Vermont reports the highest AAMR (0.543; 95% CI: 0.4101–0.7051), followed by New Mexico (0.4564; 95% CI: 0.3815–0.5313), the District of Columbia at (0.4481; 95% CI: 0.3155–0.6177), and Alaska at (0.4534; 95% CI: 0.3013–0.6553). By contrast, New Jersey shows the lowest rate at (0.2279; 95% CI: 0.2032–0.2527), with Louisiana (0.2524; 95% CI: 0.2132–0.2916), Michigan (0.2609; 95% CI: 0.2349–0.2869), and Florida (0.2642; 95% CI: 0.2466–0.2818) also on the lower end (Figure 4) (Supporting Information S1: Table S4).

**FIGURE 5:**
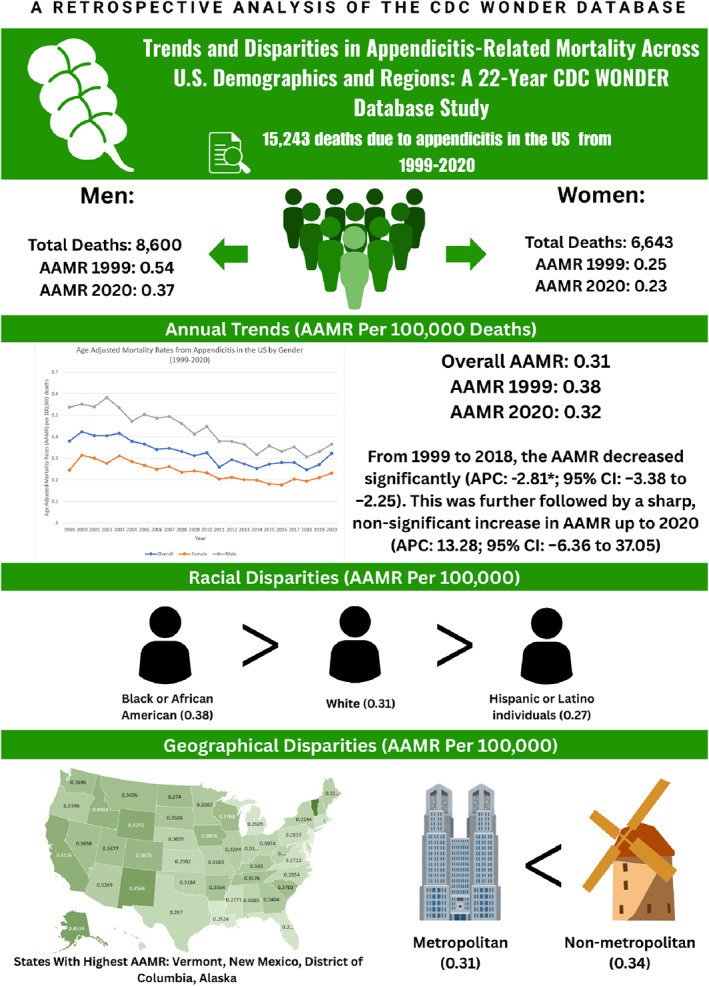
Central illustration showing summary of results.

Figure 5 shows a summary of our results.

## 4 Discussion

Our analysis recorded an overall fall in mortality rates from 1999 to 2020 despite an insignificant rise from 2018. Further, trends indicated a sharp rise in mortality among females since 2016, whereas males noted a linear decline in mortality since 1999. By race, highest AAMRs in 2020 were recorded in African Americans followed by Whites and Hispanics. Despite these rates, trends indicated a uniform decline in AAMR for each age group since 1999. Nonmetropolitan regions had the highest AAMRs in 2020. Finally, although Vermont and New Mexico noted the highest burden of mortality, New Jersey and Louisiana recorded least AAMRs in 2020.

Appendicitis‐related deaths mainly arise from perforation of the appendix. This is a surgical emergency and requires immediate intervention in the form of appendectomy, either laparoscopic or open. Our analysis indicated nearly a 1.5 time fall in mortality rates since 1999. Significant advances in the field of management of acute appendicitis, including transition from open to laparoscopic surgery, resulting in improvement in postsurgical outcomes, have significantly contributed to reducing AAMRs [22]. Data from the Nationwide Inpatient Sample indicate a significant rise in the rates of laparoscopic appendectomy since 2004, with a 100% increase in use for perforated appendicitis [23]. Laparoscopic appendectomy (LA) became widely used in the 1990s and has since been associated with shorter hospital stays, fewer wound infections, and faster recovery [24]. This advancement has greatly contributed to effective management of patients, thereby reducing appendicitis‐related morbidity and mortality over time. Moreover, changes in healthcare funding policies have also been observed over the years. The 2010 Affordable Care Act (ACA) and Medicaid expansions dramatically increased coverage. Uninsured rates were found to decrease by 3.6% among 19–25‐year‐old after the ACA Dependent Coverage Provision (letting young adults stay on family insurance). This coincided with a 1.4‐point drop in perforated appendicitis in this sub‐group, therefore highlighting the role of insurance gain in ensuring earlier diagnosis and treatment of these patients [25]. Major trials, such as the Finnish APPAC trial (2015), have shown nonoperative management is viable for many uncomplicated cases of appendicitis [26]. It was found that 73% and 61% treated with antibiotics avoided surgery at 1 and 5 years, respectively. These findings have helped establish antibiotics as a legitimate first‐line treatment option for uncomplicated appendicitis, potentially reducing the need for surgery and lowering the risk of surgery‐related and appendicitis‐related complications. Additionally, better response of the emergency medical services and immediate access to hospitals may have further contributed to the observed fall in mortality [27]. Despite this fall, an insignificant rising trend was noted since 2018. The COVID‐19 pandemic may have influenced trends only from the late 2019 and early 2020s. Although our data does not directly capture delays in care, studies have shown that delayed access to emergency services and timely healthcare intervention was a problem that was faced during the COVID‐19 pandemic [28]. This may have contributed to the small rise in mortality observed during this time period. Additionally, several studies reported higher rates of complicated (e.g. perforated) appendicitis in the pandemic's early phase [29]. These factors together may explain the slight uptick in appendicitis‐related mortality during the early phase of the pandemic, highlighting the critical importance of timely access to emergency care.

Additionally, although males noted a uniform decline in mortality, females experienced a significant surge in death rates since 2016. Studies conducted elsewhere indicate that although males have a higher risk of appendicitis, females were associated with a higher risk of negative appendectomy, often obscured by other diagnoses such as ovarian cysts or ruptured Graafian follicles [30]. Coupled with this, although global trends indicate a fall in mortality and morbidity, fall in disease‐associated life years was lesser in females than males [31]. Moreover, literature reports that misdiagnosis rates in women are high, further hindering timely intervention [32]. Response time and experience of care significantly varies in emergency departments (EDs) across different sexes. Chen et al. noted fewer women received timely care and adequate information on their health status [33]. Specific to acute abdominal pain, women received analgesics much later than men and had to wait longer before receiving a second dose according to a study by Hayes et al. [34]. This highlights a concerning sex disparity that exists in the US healthcare system. Therefore, in addition to the misdiagnosis, the disparity in care received by women in EDs calls for global action to bridge the gap in health equity across all sexes. Our results showed that women experienced overall lower mortality rates, despite known delays in diagnosis. This may be attributable to positive health‐seeking behavior and biological advantages. Literature has widely reported that women tend to utilize healthcare more than men [35]. This increased engagement may facilitate timely and effective management of diseases before complications arise, which could partly account for the lower mortality rates observed. Additionally, female biological hormones have been shown to enhance cell‐mediated, contrary to male hormones that tend to suppress immune function. Moreover, the male sex itself has also been identified as a major risk factor for postoperative infections [36]. In summary, greater utilization of healthcare services and an immunological “female advantage” may potentially explain the survival benefit seen in women for appendicitis.

Despite all races recording a decline in mortality rates since 1999, African Americans recorded the highest AAMRs. This may stem from disparities in receipt of treatment of Black patients in the ED. Black individuals were nearly 1.3 times more likely to die in the ED and were associated with lesser odds of being admitted or receiving an immediate or emergent score on first look [37]. Additionally, Black individuals had a higher risk of delayed diagnosis of appendicitis and had a higher risk of postoperative readmission [38]. Consequently, rates of postoperative complications are 1.4 times higher than White individuals [12]. This shows that although the declining trend indicates improvement in access to equitable healthcare possibly due to Medicaid [39], racial disparities still exist, calling for immediate action by the healthcare system to ensure equitable services to every patient. Interestingly, we observed lowest mortality rates among Hispanic individuals. This contradicts the general perception that Hispanic individuals are susceptible to higher mortality rates compared to Whites due to socioeconomic disadvantages. This may be explained by the well‐known “Hispanic paradox” which states that U.S. Hispanics often have equal or better survival despite socioeconomic disadvantages [40]. This may be attributed to selective (healthy) immigration and strong familial/social networks [41]. Additionally, a possible hypothesis for the low mortality observed among Hispanics may be partly attributed to the under‐reporting of appendicitis‐related deaths, potentially due to limited access to healthcare services in this population. Further studies to investigate potential reasons behind these mortality trends is recommended.

Metropolitan and nonmetropolitan regions recorded a declining trend; however, AAMRs were higher in nonmetropolitan or rural regions. The declining trends may be explained by the expansion of emergency medical services (EMS) [42]. Furthermore, Medicaid expansion and rise of insurance coverage have helped bridge the gap between rural and nonrural households allowing equitable access to healthcare [43]. Despite developments in policies and available facilities, the higher mortality rates in rural regions possibly indicate a gap in access to immediate ED services. Alruwaili et al. noted a globally higher prehospital time interval in rural regions [44]. Additionally, response times of the EMS is longer in rural regions [45]. Rural patients were also found to be more likely to present with perforation, and 30% of these patients necessitated transfer to an urban hospital for treatment [46]. Thus, these reflect the inadequate resources at rural hospitals to manage complicated cases including those with perforated appendix, leading to higher mortality rates.

Vermont and New Mexico noted the highest AAMRs. New Mexico has one of the highest rates of uninsured patients, which may result in delayed care due to costs [47]. Vermont is a predominantly rural state, with lower number of hospitals and emergency healthcare facilities [48]. New Jersey and Louisiana had the lowest AAMRs, which may have been influenced by their strong healthcare delivery system with transport networks and established emergency medical care services [49, 50]. New Jersey was also one of the states that came under Medicaid expansion in 2014, further resulting in increased insurance coverage [51].

This study summarized key demographic trends in appendicitis‐related deaths. Limitations of this study include unreliable data in certain variables due to fewer cases of deaths and the use of data from death certificates, wherein the cause of death may be incorrectly classified. Our study is limited by its reliance on CDC WONDER death certificate data, which does not include clinical variables such as time from symptom onset to diagnosis, time to surgical intervention, or in‐hospital management details. As such, any discussion of delayed care, diagnostic uncertainty, or access‐related disparities must be interpreted as hypothesis‐generating rather than conclusive. Although we observed mortality differences across sex and racial/ethnic groups, the absence of time‐to‐care metrics prevents us from directly linking these outcomes to diagnostic or treatment delays. This limitation reflects an inherent constraint of the CDC WONDER database and underscores the need for future research using more granular clinical data to confirm these correlations. Factors influencing trends, including rates of appendicitis, rates of conversion from laparoscopic to open appendectomy, and insurance coverage, were not assessed. Future studies must attempt to identify factors contributing to the slight rise in mortality since 2018. Efforts must be undertaken for policies to bring down costs of routine procedures, such as laparoscopic appendectomy, to allow for better coverage.

## 5 Conclusion

The study noted a fall in mortality due to appendicitis from 1999 to 2020. Despite the fall, females noted a significant surge in deaths since 2016. Further, African Americans noted the highest AAMRs despite declining trends, whereas rural regions consistently recorded higher mortality rates than urban regions. Further studies are needed to characterize the factors accounting for these trends. Additionally, policies should focus on bridging the gap in surgical disparities and ensure timely and safe surgical intervention for all.

## Author Contributions

**Rayyan Nabi:** conceptualization, methodology, formal analysis. **Shree Rath:** writing – original draft. **Sohaib Aftab Ahmad Chaudhry:** writing – original draft, data curation. **Najaf Ahmed Rajpar:** writing – original draft. **Sabahat Ul Ain Munir Abbasi:** writing – original draft. **Tabeer Zahid:** resources, writing – review and editing, data curation. **Raheel Ahmed:** project administration, writing – review and editing, funding acquisition.

## Ethics Statement

Ethics approval is deemed unnecessary according to national regulations as our study did not involve any human/animal test subject (http://nbcpakistan.org.pk/nbc‐r.html).

## Consent

The need for consent to participate is deemed unnecessary according to national regulations as our study did not involve any human/animal test subject (http://nbcpakistan.org.pk/nbc‐r.html). We ensured adherence to the ethical principles outlined in the Declaration of Helsinki. This has been clearly stated under the Declarations section in the manuscript.

## Conflicts of Interest

The authors declare no conflicts of interest.

## Supporting information

Supporting Information S1

## Data Availability

Data is provided within the manuscript or supplementary information files. Any extra data required will be provided on further request to the author.
