# Supplementary material for: Trends and Disparities in Appendicitis‐Related Mortality Across U.S. Demographics and Regions: A 22‐Year CDC WONDER Database Study
Source: World J Surg. 2025 Jul 28;49(9):2399–408. doi: 10.1002/wjs.70023 (PMC12435609; doi:10.1002/wjs.70023)
Supplement: Supplementary file 1 — Supporting Information S1 [file WJS-49-2399-s001.docx]

**Supplementary Table 1:** *Age-Adjusted Mortality Rates (AAMR) Related to Appendicitis in the US by Sex, 1999-2020*

| **Sex** | **Year** | **Deaths** | **Population** | **Age Adjusted Rate** | **Age Adjusted Rate Lower 95% Confidence Interval** | **Age Adjusted Rate Upper 95% Confidence Interval** | **Age Adjusted Rate Standard Error** |
| --- | --- | --- | --- | --- | --- | --- | --- |
| Overall | 1999 | 662 | 180408769 | 0.38 | 0.3507 | 0.4093 | 0.015 |
| Overall | 2000 | 735 | 181984640 | 0.4232 | 0.3923 | 0.4542 | 0.0158 |
| Overall | 2001 | 726 | 184305128 | 0.4052 | 0.3753 | 0.4351 | 0.0153 |
| Overall | 2002 | 759 | 186208028 | 0.4046 | 0.3755 | 0.4337 | 0.0148 |
| Overall | 2003 | 750 | 188090429 | 0.4163 | 0.3863 | 0.4464 | 0.0153 |
| Overall | 2004 | 698 | 190205384 | 0.3791 | 0.3501 | 0.4081 | 0.0148 |
| Overall | 2005 | 727 | 192551384 | 0.3657 | 0.3385 | 0.3929 | 0.0139 |
| Overall | 2006 | 724 | 195019359 | 0.3411 | 0.3157 | 0.3666 | 0.013 |
| Overall | 2007 | 736 | 197403777 | 0.3465 | 0.3209 | 0.3721 | 0.0131 |
| Overall | 2008 | 689 | 199795090 | 0.3315 | 0.3059 | 0.3572 | 0.0131 |
| Overall | 2009 | 674 | 202107016 | 0.3122 | 0.2882 | 0.3363 | 0.0123 |
| Overall | 2010 | 701 | 203891983 | 0.3261 | 0.3006 | 0.3516 | 0.013 |
| Overall | 2011 | 623 | 206592936 | 0.2586 | 0.2373 | 0.28 | 0.0109 |
| Overall | 2012 | 647 | 208826037 | 0.2936 | 0.2701 | 0.317 | 0.012 |
| Overall | 2013 | 636 | 211085314 | 0.2741 | 0.2513 | 0.2969 | 0.0116 |
| Overall | 2014 | 608 | 213809280 | 0.2533 | 0.2325 | 0.2742 | 0.0107 |
| Overall | 2015 | 647 | 216553817 | 0.2729 | 0.2507 | 0.2951 | 0.0113 |
| Overall | 2016 | 625 | 218641417 | 0.2805 | 0.2569 | 0.304 | 0.012 |
| Overall | 2017 | 710 | 221447331 | 0.2807 | 0.2586 | 0.3028 | 0.0113 |
| Overall | 2018 | 658 | 223311190 | 0.2464 | 0.2264 | 0.2664 | 0.0102 |
| Overall | 2019 | 688 | 224981167 | 0.2714 | 0.2499 | 0.2928 | 0.0109 |
| Overall | 2020 | 820 | 226635013 | 0.3229 | 0.2996 | 0.3461 | 0.0119 |
| Female | 1999 | 276 | 94123092 | 0.2451 | 0.2153 | 0.2749 | 0.0152 |
| Female | 2000 | 327 | 94864102 | 0.3144 | 0.2793 | 0.3494 | 0.0179 |
| Female | 2001 | 318 | 95984408 | 0.3005 | 0.2664 | 0.3346 | 0.0174 |
| Female | 2002 | 309 | 96927703 | 0.2771 | 0.2455 | 0.3086 | 0.0161 |
| Female | 2003 | 328 | 97893297 | 0.3115 | 0.2761 | 0.347 | 0.0181 |
| Female | 2004 | 322 | 98921524 | 0.2844 | 0.2528 | 0.316 | 0.0161 |
| Female | 2005 | 325 | 100097839 | 0.2682 | 0.2382 | 0.2982 | 0.0153 |
| Female | 2006 | 305 | 101328442 | 0.2489 | 0.2198 | 0.278 | 0.0148 |
| Female | 2007 | 297 | 102513830 | 0.2612 | 0.2297 | 0.2926 | 0.0161 |
| Female | 2008 | 299 | 103688156 | 0.2354 | 0.2077 | 0.2631 | 0.0141 |
| Female | 2009 | 285 | 104834186 | 0.241 | 0.2116 | 0.2704 | 0.015 |
| Female | 2010 | 297 | 105717426 | 0.233 | 0.2056 | 0.2604 | 0.014 |
| Female | 2011 | 279 | 107004480 | 0.2039 | 0.1793 | 0.2284 | 0.0125 |
| Female | 2012 | 285 | 108088829 | 0.2119 | 0.186 | 0.2378 | 0.0132 |
| Female | 2013 | 284 | 109172782 | 0.2006 | 0.1763 | 0.2249 | 0.0124 |
| Female | 2014 | 280 | 110581619 | 0.1993 | 0.1751 | 0.2235 | 0.0124 |
| Female | 2015 | 264 | 111947362 | 0.1813 | 0.1578 | 0.2048 | 0.012 |
| Female | 2016 | 263 | 112990913 | 0.1771 | 0.1549 | 0.1994 | 0.0114 |
| Female | 2017 | 317 | 114358026 | 0.2036 | 0.1803 | 0.2269 | 0.0119 |
| Female | 2018 | 293 | 115265998 | 0.1945 | 0.1715 | 0.2175 | 0.0117 |
| Female | 2019 | 313 | 116084432 | 0.2104 | 0.1864 | 0.2345 | 0.0123 |
| Female | 2020 | 377 | 116909132 | 0.2312 | 0.2071 | 0.2553 | 0.0123 |
| Male | 1999 | 386 | 86285677 | 0.5372 | 0.4819 | 0.5925 | 0.0282 |
| Male | 2000 | 408 | 87120538 | 0.5511 | 0.4965 | 0.6058 | 0.0279 |
| Male | 2001 | 408 | 88320720 | 0.5389 | 0.4852 | 0.5927 | 0.0274 |
| Male | 2002 | 450 | 89280325 | 0.5817 | 0.5267 | 0.6368 | 0.0281 |
| Male | 2003 | 422 | 90197132 | 0.5333 | 0.4813 | 0.5852 | 0.0265 |
| Male | 2004 | 376 | 91283860 | 0.4719 | 0.4227 | 0.5212 | 0.0251 |
| Male | 2005 | 402 | 92453545 | 0.5031 | 0.4529 | 0.5534 | 0.0256 |
| Male | 2006 | 419 | 93690917 | 0.4858 | 0.4379 | 0.5337 | 0.0244 |
| Male | 2007 | 439 | 94889947 | 0.4939 | 0.4464 | 0.5414 | 0.0243 |
| Male | 2008 | 390 | 96106934 | 0.4606 | 0.4141 | 0.5071 | 0.0237 |
| Male | 2009 | 389 | 97272830 | 0.4126 | 0.3701 | 0.4552 | 0.0217 |
| Male | 2010 | 404 | 98174557 | 0.4473 | 0.4024 | 0.4923 | 0.0229 |
| Male | 2011 | 344 | 99588456 | 0.3792 | 0.3384 | 0.42 | 0.0208 |
| Male | 2012 | 362 | 100737208 | 0.3788 | 0.3387 | 0.4188 | 0.0204 |
| Male | 2013 | 352 | 101912532 | 0.3642 | 0.3249 | 0.4035 | 0.02 |
| Male | 2014 | 328 | 103227661 | 0.3169 | 0.2813 | 0.3525 | 0.0182 |
| Male | 2015 | 383 | 104606455 | 0.3576 | 0.3206 | 0.3947 | 0.0189 |
| Male | 2016 | 362 | 105650504 | 0.3316 | 0.2959 | 0.3672 | 0.0182 |
| Male | 2017 | 393 | 107089305 | 0.3525 | 0.3162 | 0.3888 | 0.0185 |
| Male | 2018 | 365 | 108045192 | 0.3064 | 0.2738 | 0.3391 | 0.0166 |
| Male | 2019 | 375 | 108896735 | 0.3304 | 0.2952 | 0.3656 | 0.018 |
| Male | 2020 | 443 | 109725881 | 0.3655 | 0.3302 | 0.4008 | 0.018 |

**Supplementary Figure 1:** *Age-adjusted Mortality Rates’ Annual Percentage Change (APC) Related to Appendicitis in the US by Sex, 1999-2020*


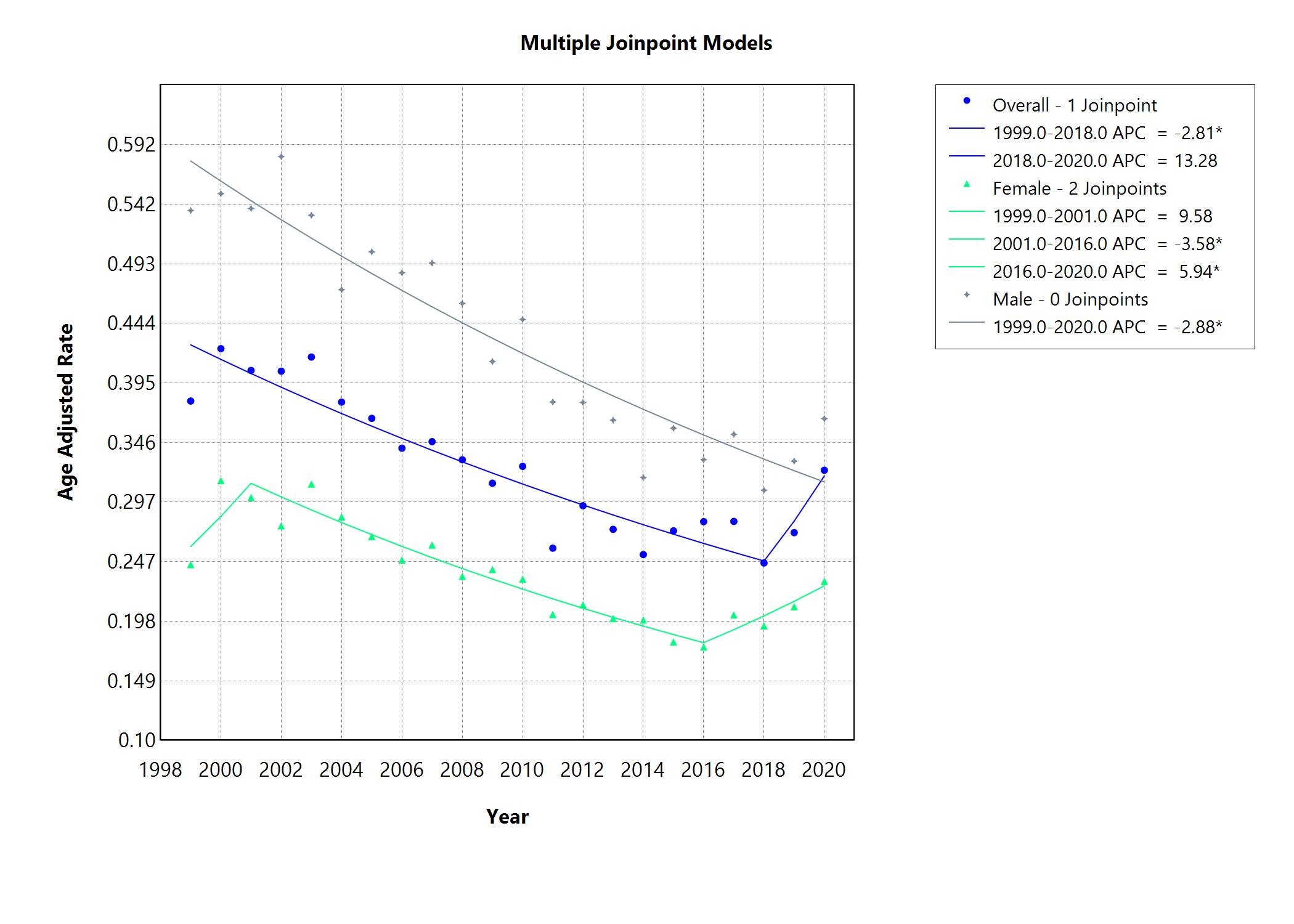


**Supplementary Table 2:** *Age-Adjusted Mortality Rates (AAMR) Related to Appendicitis in in the US by Race, 1999-2020*

| **Race** | **Year** | **Deaths** | **Population** | **Age Adjusted Rate** | **Age Adjusted Rate Lower 95% Confidence Interval** | **Age Adjusted Rate Upper 95% Confidence Interval** | **Age Adjusted Rate Standard Error** |
| --- | --- | --- | --- | --- | --- | --- | --- |
| Black or African American | 1999 | 100 | 20503656 | 0.6018 | 0.4815 | 0.7221 | 0.0614 |
| Black or African American | 2000 | 104 | 20775703 | 0.6281 | 0.5046 | 0.7517 | 0.063 |
| Black or African American | 2001 | 109 | 21181572 | 0.6041 | 0.487 | 0.7212 | 0.0597 |
| Black or African American | 2002 | 105 | 21522362 | 0.5984 | 0.4813 | 0.7155 | 0.0597 |
| Black or African American | 2003 | 93 | 21858880 | 0.4907 | 0.3935 | 0.6045 | 0.0523 |
| Black or African American | 2004 | 81 | 22258267 | 0.4452 | 0.3513 | 0.5564 | 0.0509 |
| Black or African American | 2005 | 78 | 22690893 | 0.4161 | 0.3262 | 0.5232 | 0.0487 |
| Black or African American | 2006 | 83 | 23137867 | 0.4088 | 0.3221 | 0.5116 | 0.0468 |
| Black or African American | 2007 | 104 | 23577304 | 0.5055 | 0.4048 | 0.6061 | 0.0513 |
| Black or African American | 2008 | 80 | 24018935 | 0.4052 | 0.3192 | 0.5072 | 0.0465 |
| Black or African American | 2009 | 69 | 24456946 | 0.3235 | 0.2491 | 0.413 | 0.0404 |
| Black or African American | 2010 | 73 | 24802842 | 0.3709 | 0.2886 | 0.4694 | 0.0448 |
| Black or African American | 2011 | 79 | 25288869 | 0.3425 | 0.2675 | 0.432 | 0.0408 |
| Black or African American | 2012 | 87 | 25724605 | 0.3856 | 0.3053 | 0.4806 | 0.0433 |
| Black or African American | 2013 | 67 | 26175234 | 0.2962 | 0.2261 | 0.3813 | 0.0382 |
| Black or African American | 2014 | 85 | 26742836 | 0.366 | 0.2902 | 0.4555 | 0.041 |
| Black or African American | 2015 | 63 | 27316901 | 0.2604 | 0.1973 | 0.3374 | 0.0344 |
| Black or African American | 2016 | 68 | 27833992 | 0.2455 | 0.1878 | 0.3153 | 0.0315 |
| Black or African American | 2017 | 90 | 28455786 | 0.3562 | 0.2833 | 0.4421 | 0.0393 |
| Black or African American | 2018 | 77 | 28919914 | 0.2763 | 0.2154 | 0.3491 | 0.033 |
| Black or African American | 2019 | 63 | 29385485 | 0.2492 | 0.1888 | 0.3229 | 0.033 |
| Black or African American | 2020 | 106 | 29818678 | 0.36 | 0.2885 | 0.4314 | 0.0365 |
| White | 1999 | 549 | 151306036 | 0.3596 | 0.3291 | 0.3901 | 0.0156 |
| White | 2000 | 617 | 152222052 | 0.395 | 0.3634 | 0.4266 | 0.0161 |
| White | 2001 | 598 | 153608264 | 0.3709 | 0.3407 | 0.401 | 0.0154 |
| White | 2002 | 636 | 154739951 | 0.3989 | 0.3674 | 0.4304 | 0.0161 |
| White | 2003 | 632 | 155864104 | 0.4002 | 0.3681 | 0.4322 | 0.0163 |
| White | 2004 | 596 | 157144830 | 0.354 | 0.3249 | 0.3831 | 0.0149 |
| White | 2005 | 631 | 158595744 | 0.3705 | 0.3408 | 0.4002 | 0.0151 |
| White | 2006 | 622 | 160142968 | 0.3435 | 0.316 | 0.371 | 0.014 |
| White | 2007 | 603 | 161626163 | 0.3489 | 0.3205 | 0.3774 | 0.0145 |
| White | 2008 | 585 | 163116422 | 0.3422 | 0.3135 | 0.3708 | 0.0146 |
| White | 2009 | 575 | 164541687 | 0.3146 | 0.2884 | 0.3409 | 0.0134 |
| White | 2010 | 599 | 165644483 | 0.3285 | 0.301 | 0.356 | 0.0141 |
| White | 2011 | 517 | 167382563 | 0.2402 | 0.2187 | 0.2617 | 0.011 |
| White | 2012 | 535 | 168663175 | 0.2723 | 0.2478 | 0.2967 | 0.0125 |
| White | 2013 | 546 | 169982244 | 0.2765 | 0.2517 | 0.3013 | 0.0127 |
| White | 2014 | 499 | 171489057 | 0.2509 | 0.2276 | 0.2743 | 0.0119 |
| White | 2015 | 550 | 173031552 | 0.2864 | 0.2613 | 0.3115 | 0.0128 |
| White | 2016 | 538 | 174238249 | 0.2644 | 0.2407 | 0.2882 | 0.0121 |
| White | 2017 | 594 | 175747095 | 0.2807 | 0.2565 | 0.3049 | 0.0124 |
| White | 2018 | 555 | 176757620 | 0.2533 | 0.231 | 0.2756 | 0.0114 |
| White | 2019 | 589 | 177619677 | 0.2738 | 0.25 | 0.2976 | 0.0121 |
| White | 2020 | 677 | 178513471 | 0.3044 | 0.2808 | 0.328 | 0.012 |
| Hispanic or Latino | 1999 | 34 | 17503631 | 0.312 | 0.2073 | 0.451 | 0.0595 |
| Hispanic or Latino | 2000 | 33 | 18219679 | 0.3154 | 0.2096 | 0.4558 | 0.0594 |
| Hispanic or Latino | 2001 | 36 | 19290018 | 0.3129 | 0.2111 | 0.4467 | 0.0569 |
| Hispanic or Latino | 2002 | 35 | 20159630 | 0.3239 | 0.2185 | 0.4624 | 0.0588 |
| Hispanic or Latino | 2003 | 48 | 21011656 | 0.4016 | 0.2869 | 0.5469 | 0.0633 |
| Hispanic or Latino | 2004 | 54 | 21877214 | 0.3836 | 0.2798 | 0.5132 | 0.0569 |
| Hispanic or Latino | 2005 | 35 | 22804023 | 0.2437 | 0.1632 | 0.3499 | 0.0456 |
| Hispanic or Latino | 2006 | 45 | 23743864 | 0.3238 | 0.2324 | 0.4392 | 0.0506 |
| Hispanic or Latino | 2007 | 45 | 24673919 | 0.277 | 0.197 | 0.3787 | 0.0444 |
| Hispanic or Latino | 2008 | 50 | 25602850 | 0.3015 | 0.2182 | 0.4062 | 0.0462 |
| Hispanic or Latino | 2009 | 63 | 26504021 | 0.341 | 0.2562 | 0.4449 | 0.0466 |
| Hispanic or Latino | 2010 | 43 | 27192663 | 0.2194 | 0.1528 | 0.3051 | 0.0371 |
| Hispanic or Latino | 2011 | 54 | 28255675 | 0.2683 | 0.1957 | 0.359 | 0.0401 |
| Hispanic or Latino | 2012 | 57 | 28988437 | 0.2884 | 0.2119 | 0.3835 | 0.0419 |
| Hispanic or Latino | 2013 | 45 | 29784174 | 0.2096 | 0.1468 | 0.2902 | 0.0348 |
| Hispanic or Latino | 2014 | 57 | 30809714 | 0.242 | 0.1796 | 0.3191 | 0.0342 |
| Hispanic or Latino | 2015 | 45 | 31761872 | 0.1682 | 0.1178 | 0.2328 | 0.0281 |
| Hispanic or Latino | 2016 | 57 | 32438262 | 0.2427 | 0.1812 | 0.3182 | 0.0337 |
| Hispanic or Latino | 2017 | 56 | 33594503 | 0.2214 | 0.1638 | 0.2927 | 0.0316 |
| Hispanic or Latino | 2018 | 75 | 34350362 | 0.2692 | 0.2064 | 0.345 | 0.0343 |
| Hispanic or Latino | 2019 | 69 | 35025850 | 0.242 | 0.1843 | 0.3122 | 0.0316 |
| Hispanic or Latino | 2020 | 71 | 35758193 | 0.2471 | 0.1899 | 0.3162 | 0.0311 |

**Supplementary Figure 2:** *Age-adjusted Mortality Rates’ Annual Percentage Change (APC) Related to Appendicitis in the US by Race, 1999-2020*


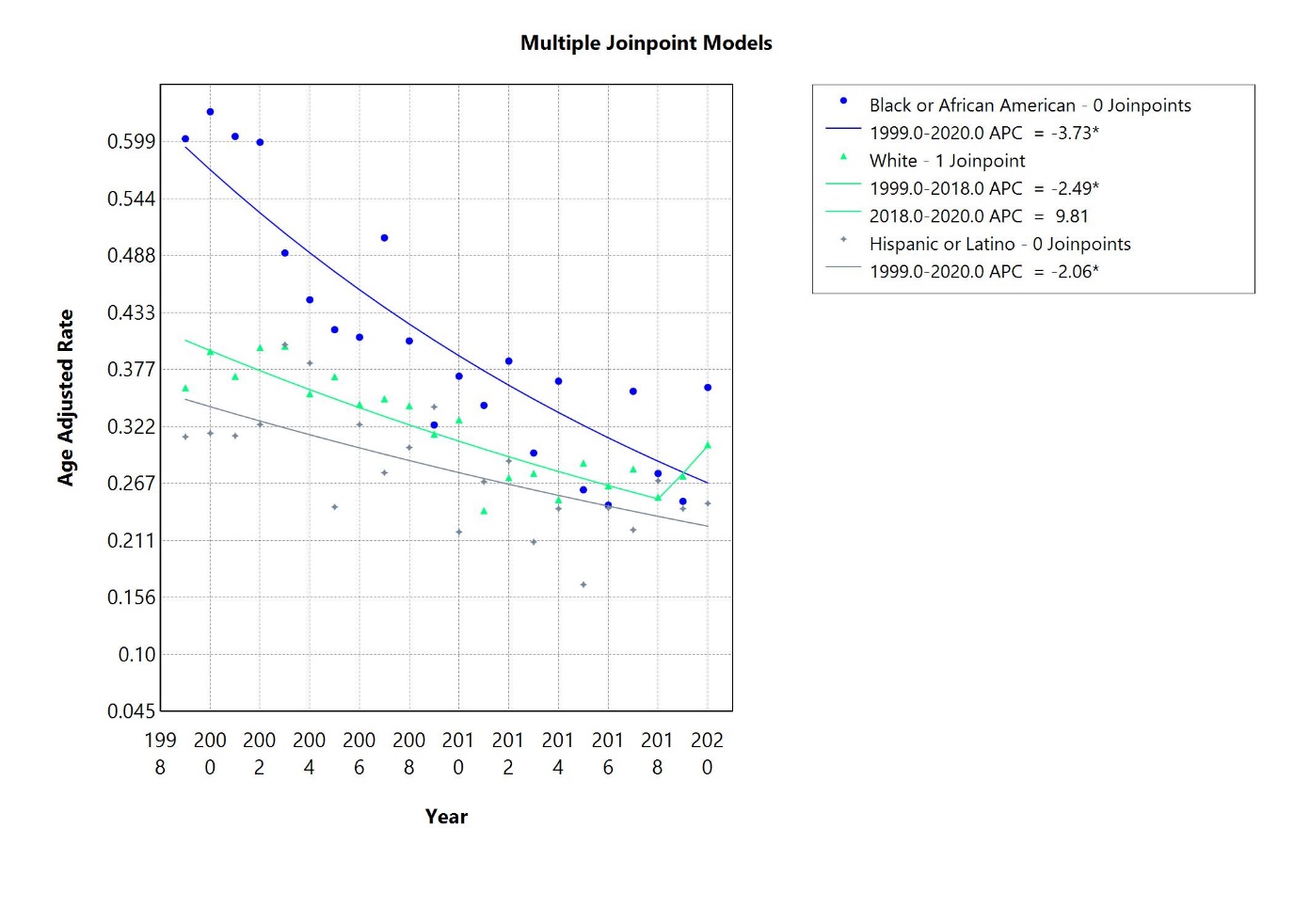


**Supplementary Table 3:** *Age-Adjusted Mortality Rates (AAMR) Related to Appendicitis in the US by Metropolitan Status, 1999-2020*

| **Urbanization** | **Year** | **Deaths** | **Population** | **Age Adjusted Rate** | **Age Adjusted Rate Lower 95% Confidence Interval** | **Age Adjusted Rate Upper 95% Confidence Interval** | **Age Adjusted Rate Standard Error** |
| --- | --- | --- | --- | --- | --- | --- | --- |
| Metropolitan | 1999 | 542 | 151245342 | 0.3683 | 0.3369 | 0.3997 | 0.016 |
| Metropolitan | 2000 | 614 | 152658699 | 0.4163 | 0.383 | 0.4496 | 0.017 |
| Metropolitan | 2001 | 583 | 154896258 | 0.3774 | 0.3462 | 0.4086 | 0.0159 |
| Metropolitan | 2002 | 602 | 156659051 | 0.3998 | 0.3675 | 0.4321 | 0.0165 |
| Metropolitan | 2003 | 596 | 158367715 | 0.3968 | 0.3646 | 0.429 | 0.0164 |
| Metropolitan | 2004 | 547 | 160272097 | 0.3296 | 0.3011 | 0.3581 | 0.0145 |
| Metropolitan | 2005 | 584 | 162371826 | 0.3585 | 0.3287 | 0.3883 | 0.0152 |
| Metropolitan | 2006 | 594 | 164523389 | 0.3543 | 0.3253 | 0.3834 | 0.0148 |
| Metropolitan | 2007 | 596 | 166650886 | 0.3548 | 0.3257 | 0.3838 | 0.0148 |
| Metropolitan | 2008 | 567 | 168826027 | 0.3492 | 0.3195 | 0.3789 | 0.0151 |
| Metropolitan | 2009 | 542 | 170965574 | 0.3074 | 0.2809 | 0.334 | 0.0135 |
| Metropolitan | 2010 | 542 | 172591105 | 0.2972 | 0.2707 | 0.3237 | 0.0135 |
| Metropolitan | 2011 | 497 | 175204532 | 0.2538 | 0.2304 | 0.2773 | 0.012 |
| Metropolitan | 2012 | 516 | 177423676 | 0.2842 | 0.2588 | 0.3097 | 0.013 |
| Metropolitan | 2013 | 524 | 179634449 | 0.2672 | 0.2426 | 0.2918 | 0.0126 |
| Metropolitan | 2014 | 497 | 182304016 | 0.2407 | 0.2187 | 0.2627 | 0.0112 |
| Metropolitan | 2015 | 524 | 184959306 | 0.2738 | 0.2492 | 0.2984 | 0.0126 |
| Metropolitan | 2016 | 494 | 186963190 | 0.2117 | 0.192 | 0.2313 | 0.01 |
| Metropolitan | 2017 | 570 | 189675062 | 0.2714 | 0.2475 | 0.2953 | 0.0122 |
| Metropolitan | 2018 | 538 | 191415024 | 0.244 | 0.2221 | 0.266 | 0.0112 |
| Metropolitan | 2019 | 555 | 193006488 | 0.2542 | 0.2317 | 0.2768 | 0.0115 |
| Metropolitan | 2020 | 683 | 194600110 | 0.3183 | 0.2933 | 0.3434 | 0.0128 |
| Non-metropolitan | 1999 | 120 | 29163427 | 0.3544 | 0.2898 | 0.419 | 0.033 |
| Non-metropolitan | 2000 | 121 | 29325941 | 0.3595 | 0.2943 | 0.4248 | 0.0333 |
| Non-metropolitan | 2001 | 143 | 29408870 | 0.453 | 0.377 | 0.529 | 0.0388 |
| Non-metropolitan | 2002 | 157 | 29548977 | 0.4778 | 0.4024 | 0.5532 | 0.0385 |
| Non-metropolitan | 2003 | 154 | 29722714 | 0.4454 | 0.374 | 0.5169 | 0.0365 |
| Non-metropolitan | 2004 | 151 | 29933287 | 0.4561 | 0.3819 | 0.5302 | 0.0378 |
| Non-metropolitan | 2005 | 143 | 30179558 | 0.4068 | 0.3386 | 0.4751 | 0.0348 |
| Non-metropolitan | 2006 | 130 | 30495970 | 0.3858 | 0.3178 | 0.4537 | 0.0347 |
| Non-metropolitan | 2007 | 140 | 30752891 | 0.387 | 0.3222 | 0.4519 | 0.0331 |
| Non-metropolitan | 2008 | 122 | 30969063 | 0.3466 | 0.2838 | 0.4093 | 0.032 |
| Non-metropolitan | 2009 | 132 | 31141442 | 0.3588 | 0.2967 | 0.4209 | 0.0317 |
| Non-metropolitan | 2010 | 159 | 31300878 | 0.4596 | 0.385 | 0.5342 | 0.038 |
| Non-metropolitan | 2011 | 126 | 31388404 | 0.3439 | 0.2815 | 0.4063 | 0.0318 |
| Non-metropolitan | 2012 | 131 | 31402361 | 0.3264 | 0.2687 | 0.384 | 0.0294 |
| Non-metropolitan | 2013 | 112 | 31450865 | 0.2958 | 0.2388 | 0.3529 | 0.0291 |
| Non-metropolitan | 2014 | 111 | 31505264 | 0.3102 | 0.2496 | 0.3708 | 0.0309 |
| Non-metropolitan | 2015 | 123 | 31594511 | 0.2975 | 0.2428 | 0.3521 | 0.0279 |
| Non-metropolitan | 2016 | 131 | 31678227 | 0.3337 | 0.2745 | 0.3929 | 0.0302 |
| Non-metropolitan | 2017 | 140 | 31772269 | 0.3669 | 0.3026 | 0.4311 | 0.0328 |
| Non-metropolitan | 2018 | 120 | 31896166 | 0.2776 | 0.2267 | 0.3284 | 0.0259 |
| Non-metropolitan | 2019 | 133 | 31974679 | 0.3147 | 0.2581 | 0.3714 | 0.0289 |
| Non-metropolitan | 2020 | 137 | 32028405 | 0.3156 | 0.2608 | 0.3704 | 0.028 |

**Supplementary Figure 3:** *Age-adjusted Mortality Rates’ Annual Percentage Change (APC) Related to Appendicitis in the US by Region, 1999-2020*


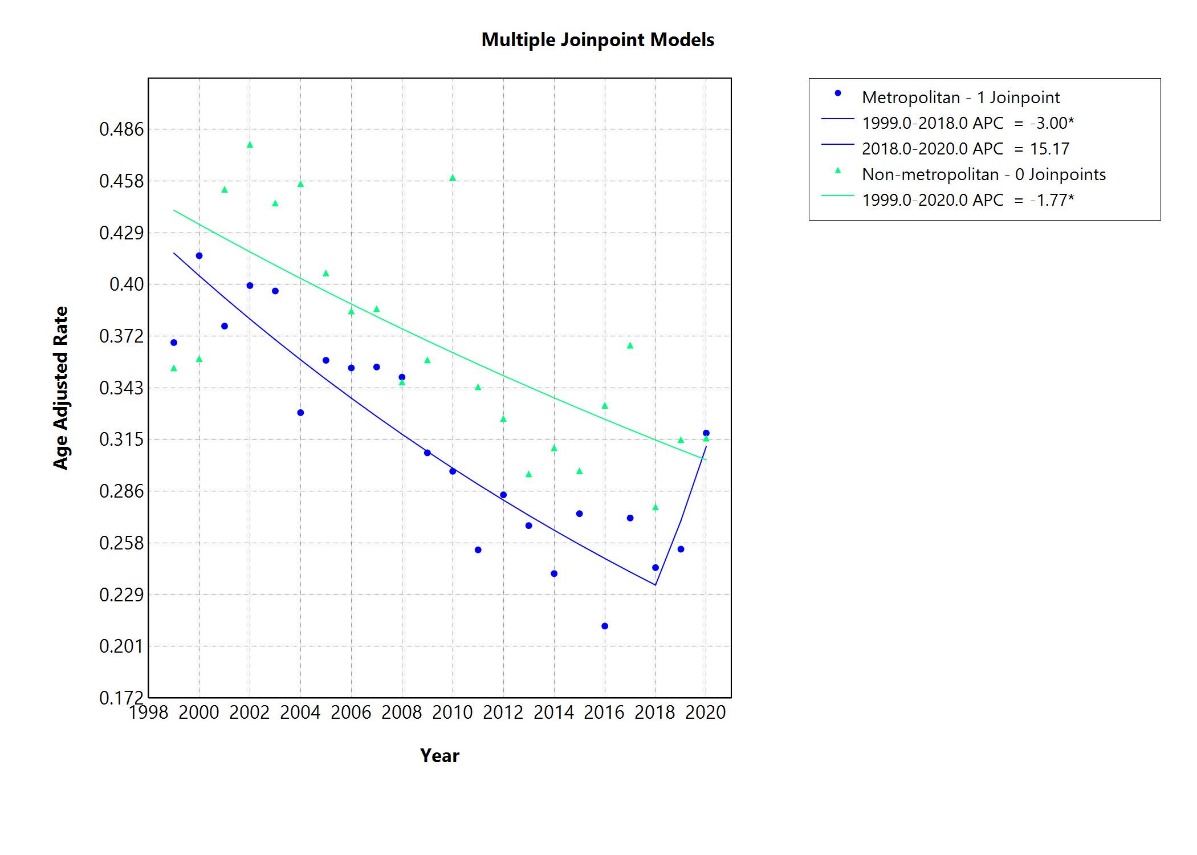


**Supplementary Table 4:** *Age-Adjusted Mortality Rates (AAMR) related to Appendicitis in the US by State, 1999-2020*

| ***State*** | ***Deaths*** | ***Population*** | ***Age Adjusted Rate*** | ***Age Adjusted Rate Lower 95% Confidence Interval*** | ***Age Adjusted Rate Upper 95% Confidence Interval*** | ***Age Adjusted Rate Standard Error*** |
| --- | --- | --- | --- | --- | --- | --- |
| *Alabama* | *227* | *68915349* | *0.3089* | *0.2677* | *0.3502* | *0.0211* |
| *Alaska* | *34* | *9623822* | *0.4534* | *0.3013* | *0.6553* | *0.086* |
| *Arizona* | *310* | *90089006* | *0.3269* | *0.2895* | *0.3643* | *0.0191* |
| *Arkansas* | *167* | *41711420* | *0.3564* | *0.3018* | *0.411* | *0.0279* |
| *California* | *2120* | *530625638* | *0.4136* | *0.3957* | *0.4314* | *0.0091* |
| *Colorado* | *248* | *73189174* | *0.3878* | *0.3386* | *0.4371* | *0.0251* |
| *Connecticut* | *158* | *53012824* | *0.2681* | *0.2236* | *0.3126* | *0.0227* |
| *Delaware* | *42* | *13210778* | *0.3019* | *0.2176* | *0.4081* | *0.0467* |
| *District of Columbia* | *38* | *9448827* | *0.4481* | *0.3155* | *0.6177* | *0.0735* |
| *Florida* | *957* | *289701852* | *0.2642* | *0.2466* | *0.2818* | *0.009* |
| *Georgia* | *416* | *135415629* | *0.3404* | *0.3066* | *0.3742* | *0.0172* |
| *Hawaii* | *66* | *20066183* | *0.2736* | *0.2098* | *0.3508* | *0.0347* |
| *Idaho* | *90* | *21392071* | *0.4004* | *0.3211* | *0.4933* | *0.0426* |
| *Illinois* | *647* | *184489320* | *0.3294* | *0.303* | *0.3558* | *0.0135* |
| *Indiana* | *280* | *92386904* | *0.3119* | *0.2738* | *0.3499* | *0.0194* |
| *Iowa* | *200* | *44030204* | *0.3876* | *0.3321* | *0.443* | *0.0283* |
| *Kansas* | *136* | *39931570* | *0.2982* | *0.2464* | *0.3501* | *0.0264* |
| *Kentucky* | *229* | *63061451* | *0.343* | *0.2973* | *0.3886* | *0.0233* |
| *Louisiana* | *170* | *64895449* | *0.2524* | *0.2132* | *0.2916* | *0.02* |
| *Maine* | *78* | *20521512* | *0.3239* | *0.2547* | *0.406* | *0.0375* |
| *Maryland* | *237* | *84902583* | *0.2595* | *0.2253* | *0.2937* | *0.0174* |
| *Massachusetts* | *307* | *99096796* | *0.2747* | *0.242* | *0.3073* | *0.0167* |
| *Michigan* | *431* | *145550610* | *0.2609* | *0.2349* | *0.2869* | *0.0133* |
| *Minnesota* | *288* | *77002440* | *0.3387* | *0.2981* | *0.3794* | *0.0207* |
| *Mississippi* | *124* | *41496865* | *0.2771* | *0.227* | *0.3273* | *0.0256* |
| *Missouri* | *298* | *86518061* | *0.3183* | *0.2799* | *0.3568* | *0.0196* |
| *Montana* | *56* | *14577633* | *0.3696* | *0.2768* | *0.4834* | *0.0509* |
| *Nebraska* | *98* | *25869503* | *0.3099* | *0.2505* | *0.3793* | *0.032* |
| *Nevada* | *134* | *38580189* | *0.3658* | *0.302* | *0.4296* | *0.0326* |
| *New Hampshire* | *63* | *19856602* | *0.3226* | *0.2468* | *0.4144* | *0.0413* |
| *New Jersey* | *346* | *130947870* | *0.2279* | *0.2032* | *0.2527* | *0.0126* |
| *New Mexico* | *146* | *28568524* | *0.4564* | *0.3815* | *0.5313* | *0.0382* |
| *New York* | *994* | *288624005* | *0.3144* | *0.2944* | *0.3344* | *0.0102* |
| *North Carolina* | *433* | *137233223* | *0.2954* | *0.2663* | *0.3246* | *0.0149* |
| *North Dakota* | *46* | *9822062* | *0.374* | *0.2718* | *0.5021* | *0.0563* |
| *Ohio* | *584* | *169651360* | *0.3074* | *0.2819* | *0.3329* | *0.013* |
| *Oklahoma* | *183* | *53110132* | *0.3184* | *0.2709* | *0.366* | *0.0243* |
| *Oregon* | *202* | *57214641* | *0.3346* | *0.286* | *0.3832* | *0.0248* |
| *Pennsylvania* | *642* | *189975429* | *0.2837* | *0.2603* | *0.3072* | *0.012* |
| *Rhode Island* | *66* | *15765032* | *0.3859* | *0.2972* | *0.4928* | *0.0483* |
| *South Carolina* | *256* | *67227226* | *0.3702* | *0.3221* | *0.4182* | *0.0245* |
| *South Dakota* | *49* | *11603220* | *0.3508* | *0.2578* | *0.4665* | *0.051* |
| *Tennessee* | *349* | *92689763* | *0.3578* | *0.3196* | *0.396* | *0.0195* |
| *Texas* | *943* | *344632461* | *0.297* | *0.2773* | *0.3168* | *0.0101* |
| *Utah* | *113* | *34057911* | *0.3677* | *0.2981* | *0.4374* | *0.0355* |
| *Vermont* | *60* | *9393709* | *0.543* | *0.4101* | *0.7051* | *0.0726* |
| *Virginia* | *331* | *116579933* | *0.2712* | *0.2408* | *0.3016* | *0.0155* |
| *Washington* | *373* | *98931323* | *0.3696* | *0.331* | *0.4083* | *0.0197* |
| *West Virginia* | *99* | *28023276* | *0.3142* | *0.2527* | *0.3862* | *0.0332* |
| *Wisconsin* | *344* | *82712057* | *0.3788* | *0.3376* | *0.4199* | *0.021* |
| *Wyoming* | *35* | *7921067* | *0.4255* | *0.2929* | *0.5975* | *0.074* |
